# Assessing environmental features related to mental health: a reliability study of visual streetscape images

**DOI:** 10.1186/1471-2458-14-1094

**Published:** 2014-10-22

**Authors:** Yu-Tzu Wu, Paul Nash, Linda E Barnes, Thais Minett, Fiona E Matthews, Andy Jones, Carol Brayne

**Affiliations:** Department of Public Health and Primary Care, Institute of Public Health, Forvie Site, University of Cambridge, School of Clinical Medicine, Cambridge Biomedical Campus, Cambridge, CB2 0SR UK; Centre for Innovative Ageing, College of Human and Health Science, Swansea University, Swansea, SA2 8PP UK; MRC Biostatistics Unit, Institute of Public Health, University of Cambridge, Cambridge, CB2 0SR UK; Norwich Medical School, University of East Anglia, Norwich, Norfolk NR4 7TJ UK

**Keywords:** Neighbourhood, Audit tool development, Mental health, Built environment, Residential environmental assessment tool

## Abstract

**Background:**

An association between depressive symptoms and features of built environment has been reported in the literature. A remaining research challenge is the development of methods to efficiently capture pertinent environmental features in relevant study settings. Visual streetscape images have been used to replace traditional physical audits and directly observe the built environment of communities. The aim of this work is to examine the inter-method reliability of the two audit methods for assessing community environments with a specific focus on physical features related to mental health.

**Methods:**

Forty-eight postcodes in urban and rural areas of Cambridgeshire, England were randomly selected from an alphabetical list of streets hosted on a UK property website. The assessment was conducted in July and August 2012 by both physical and visual image audits based on the items in Residential Environment Assessment Tool (REAT), an observational instrument targeting the micro-scale environmental features related to mental health in UK postcodes. The assessor used the images of Google Street View and virtually "walked through" the streets to conduct the property and street level assessments. Gwet’s AC1 coefficients and Bland-Altman plots were used to compare the concordance of two audits.

**Results:**

The results of conducting the REAT by visual image audits generally correspond to direct observations. More variations were found in property level items regarding physical incivilities, with broad limits of agreement which importantly lead to most of the variation in the overall REAT score. Postcodes in urban areas had lower consistency between the two methods than rural areas.

**Conclusions:**

Google Street View has the potential to assess environmental features related to mental health with fair reliability and provide a less resource intense method of assessing community environments than physical audits.

## Background

A consistent association between depressive symptoms and features of built environment has been reported in a small number of studies controlling for individual level socioeconomic characteristics [1]. Recent reviews have suggested that physical characteristics of local areas, such as green space provision, land use mix, safety and design features, may influence the mental health and well-being of residents [2, 3]. These works have questioned what the mechanism could be. A recent review proposed a causal pathway that poor characteristics of built environment encourage social disorders and crime in neighbourhoods with a potential impact on stress and a lack of control as well as increasing the risk of depression through the deterioration of supportive social networks [4]. Alternatively, it might be that social disorder mediates associations between poor community environment and mental health. Some micro-scale environmental features at the street and property level, such as littered streets, graffiti and broken windows on properties, are considered to be associated with signs of social disorder and may therefore be important risk factors for mental illnesses [5, 6]. However, the existing studies have been small with limited statistical power to measure substantial effect sizes [7–11]. Since these specific features at the property and street level need to be observed by physical audits, it is particularly difficult to conduct efficient data collection for large populations. With the development of new technology, semi-computerised systems may help address these limitations.

Traditional physical audit has involved assessors visiting local areas and rating the environment at particular times. This is subject to several unpredictable factors, such as inclement weather or poor transport links which may influence the rate of progress or the safety of assessors [12, 13]. Videotaping and photography have been used in some studies yet have been noted to be costly and time-consuming [14–18]. As a result direct community observations have been conducted using visual streetscape images, including Google Street View, Google Earth, Bing Maps or omnidirectional imagery, in the last three years [13, 15, 19–21]. The results of visual image audits generally correspond well to physical audits and have a lower resource burden than the previous techniques. However, these previous studies either examined the general quality of the built environment without reference to specific health outcomes or were focused on environmental features related to physical activity. There is the potential to apply the new measurement method of built environment to other aspects of health. To develop a valid but affordable measurement method for investigating environmental features related to mental health, the reliability of physical and visual image audits is a key issue that needs to be fully explored.

This study presents a new method using less resource intensive streetscape images to support environmental assessment in mental health studies. We examines the inter-method reliability and level of agreement between physical and visual image audits based on a standardised instrument, Residential Environment Assessment Tool (REAT), which has been used to assess specific environmental features associated with mental health and quality of life [22]. The streetscape images in Google Street View are used to conduct property and street level assessments in the urban and rural areas of the UK and explore the possibility of conducting the REAT by visual image audits in large populations.

## Methods

### Sample

This validation study was conducted mainly in Cambridgeshire, England. Twenty-four postcodes in urban areas (Cambridge city) and 24 postcodes in rural areas (towns and villages in the north of the town of Ely) were selected randomly from the alphabetical list of streets on Zoopla, a UK property websites (http://www.zoopla.co.uk). Random numbers were generated to select the street in each location. If more than one postcode unit was present in one street, another random number was generated to select the unit. The postcodes containing only colleges, departments and institutes were excluded. The definition of urban and rural areas used corresponded to the 2011 Rural–urban Classification for Small Area Geographies, an official category produced by the UK government [23].

### Measures

#### Residential Environment Assessment Tool (REAT)

REAT is an observational instrument designed to rate the quality of the living environment comprehensively for geographical units of UK postcodes. It contains 28 property and street level items in four domains of physical features that describe the street level environment. Exposure to many of these items has previously been associated with mental health outcomes and well-being in the studies referenced. Physical incivilities measure the features of neglect and tolerance of offensive behaviours, such as broken windows, graffiti, litter in the street and abandoned cars [7, 8, 24]. Territorial functioning considers non-verbal claim of control in private areas, such as external beautification and garden maintenance [25]. Defensible space focuses on environmental designs which encourage residents to take control of community, such as barriers, walls, fences and shrubbery [11]. The natural environment includes trees, green space, recreation space and aesthetics [3].

The assessment can be completed by an independent observer without relying on the perceptions of residents. The reliability and validity of REAT has been examined and confirmed in a previous study [22].

#### Google street view

Google Street View is a technology which provides 360-degree horizontal and 290-degree vertical panoramic views along many streets in the world. The images were taken from a height of about 2.5 metres at approximately 10 or 20 metres intervals by the cars or trikes with special equipment. The photos were connected and turned into a continuous panorama. It was initially started in several cities in the United States in 2007 and expanded to urban and rural areas worldwide. The public-access image data of the United Kingdom has been added since 2009 and covered most areas by 2010. The images in Cambridgeshire used in the present analysis were mainly taken in 2008.

### Procedure

**Figure 1:**
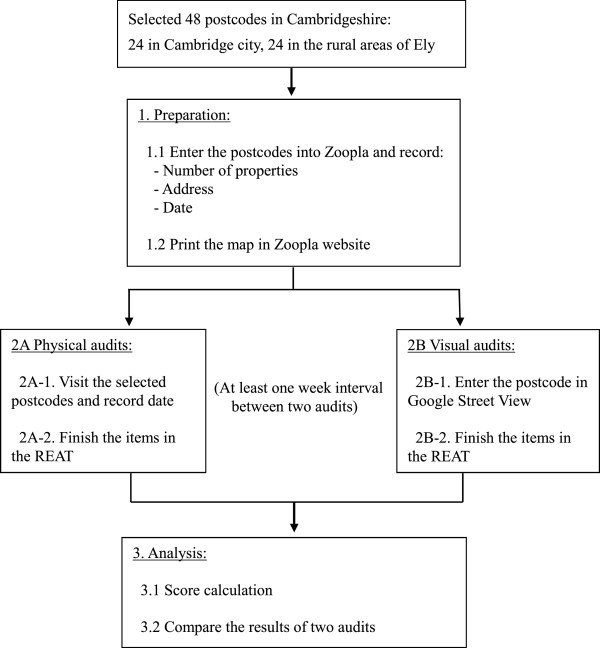
**The process of the validation study.**

The study includes three main stages: preparation, physical/visual image audits, and analysis. The study process is shown in Figure 1.

#### Preparation

In order to assess property level items in the REAT, the selected 48 postcodes were entered into Zoopla, a UK property website (http://www.zoopla.co.uk), to extract the information on house numbers and addresses of properties in the postcodes. Maps were printed to assist in determining the areas of investigation.

#### Physical/ visual image audits

Since there was only one assessor, the visual and physical audits were separated by at least one week interval to avoid contamination. Half of urban and rural postcodes were conducted by undertaking the visual image audits first and physical assessment later and the other half were counterbalanced and conducted in the opposite order. The order of assessment methods was arranged randomly.

Data collection was carried out in July and August 2012. Physical audits followed the instruction in the REAT manual. For visual image audits, the selected postcodes were firstly entered into the Google Street View and connected to the image database. The assessor virtually "walked through" the whole streets in the postcode unit and assessed the REAT items based on the visual streetscapes.

#### Analysis

Score calculation was based on the scoring system in the REAT manual. Three (0, 0.5, 1 for low/medium/high) or two (0, 1 for yes/no) categories of scores were applied to each item. Higher scores indicate more features of disorder or poorer environment. To sum up the total REAT score, the scores of each item were multiplied by the weights which ranged from 1 to 3 based on a local residential survey [22].

The extent of agreement between the two methods was assessed by calculating Gwet’s AC1 coefficients. The choice of this method was based on the fact that this is a paradox-resistant alternative to Kappa’s coefficient when the overall present agreement is high [26]. The coefficient calculations were performed using Agreestat 2011.2 software. The extent of agreement was assessed using the benchmark proposed by Landis and Koch, with a coefficient >0.6 indicating substantial agreement and a value >0.8 near-perfect agreement [27]. The levels of agreement in this study were classified into three types: almost perfect (>0.8), substantial (0.6 ~ 0.8) and fair/slight (<0.6). The difference between physical and visual image audits in the total and domain score of the REAT was examined by Bland-Altman plots [28]. To compare the concordance of the two assessment methods, the difference of the REAT score between the physical and visual image audits (the score of physical audits – the score of visual image audits) was calculated and the distribution of this difference was described by the mean, standard deviation and 95% limits of agreement (mean ±1.96 × standard deviation), which indicates the range of variation between the two methods. To provide the comparison for the total score and four domains, the magnitude of variation (1.96 × standard deviation/detected score range × 100%) was calculated considering the size of variation among the range of detected scores.

## Results

Five of the postcodes contained flats as the main type of property. Since the property level conditions of flats cannot be assessed individually, the information on property assessment in these postcodes was excluded from the analysis. In total, there were 48 data at street level and 43 data at property level.

Among the 48 postcodes, the Google Street View images were taken during the period 2008 to 2011. The visual image audits of 30 postcodes were based on the images from 2008, four from 2009, two from 2010 and two from 2011. Ten postcodes contained mixed data from 2008 and other years. The comparison of time and equipment required for data collection in the two methods are reported in Table 1. The assessments of 48 postcodes were finished in 5.5 working days by physical audits and 4.5 days by visual image audits. Visual image audits did not therefore save considerable time in data collection but could save research costs such as transport and equipment.

**Table 1:** **A comparison of time and potential costs in physical and visual image audits**

	Physical audits	Visual image audits
Working days	5.5	4.5
Transport	Bus (urban), private vehicle (rural)	None
REAT questionnaire	Paper-based	Computer-based
Data collection	Maps, stationary, camera	Internet
Data entry	Yes	No (finished in data collection)

### Comparison of total and domain scores

**Figure 2:**
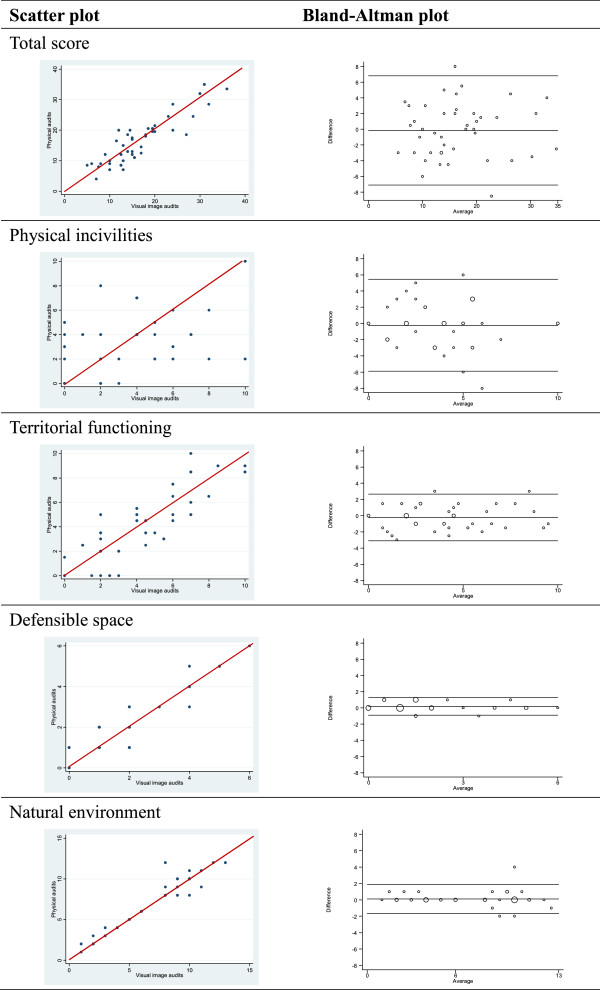
**The comparison of total and domain score in physical and visual image audits by scatter plot and Bland-Altman plot.**

Positive relationships between the physical and visual image audits were found in all four domains. Scatter plots comparing the total REAT score of the 48 study postcodes between the two methods are presented in Figure 2. In terms of the total score, the distribution of points closely followed the line of equality and showed a strong relationship. Among the four domains, the limits of agreement were narrower for the measures of defensible space and the natural environment but were relatively wider for territorial functioning and physical incivilities. This indicated that street attributes regarding defensible space and the natural environment were more consistently scored between the physical and visual image audits compared to physical incivilities and territorial functioning.

More detailed information on the difference between physical and visual image audits is provided Table 2. The range of detected score was from 4.0 to 36.0. The mean difference of total score between two methods was -0.15 and the limits of agreement were from -7.1 to 6.8. Among 95% of the postcodes, the difference of total score between the two methods was less than 7, which was about 22% of the detected score range. The broad interval of limits of agreement can be mainly attributed to the variation in physical incivilities where 95% limits of agreement were from -5.9 to 5.4. This indicates that most postcodes had a score difference up to 6, which was 57% of detected range. Compared to physical incivilities, the differences in defensible space (18%) and natural environment (14%) were smaller, indicating less variation between physical and visual image audits in these domains.

**Table 2:** **The mean difference and limits of agreement of total and domain scores**

	Max possible score	Detected score range	Mean difference (95% CI)	Limits of agreement	Magnitude of variation
Total score	68	4.00, 36.00	-0.15 (-1.22, 0.92)	-7.10, 6.80	22%
Physical incivilities	36	0.00, 10.00	-0.23 (-1.05, 0.64)	-5.90, 5.44	57%
Territorial functioning	10	0.00, 10.00	-0.22 (-0.64, 0.22)	-3.10, 2.66	29%
Defensible space	6	0.00, 06.00	0.19 (0.02, 0.35)	-0.91, 1.28	18%
Natural environment	16	1.00, 13.50	0.12 (-0.15, 0.39)	-1.64, 1.87	14%

### Agreement of the REAT items

Table 3 demonstrates the frequency of events in two audits and the Gwet’s AC1 coefficients for each item of REAT. Some features of physical incivilities such as burnt out properties, broken windows and dog litter were rare.

**Table 3:** **A comparison of REAT derived from physical or visual methods and their agreement (Property level items N = 43; Street level items N = 48)**

Domain		Question	Physical (n)	Visual (n)	Gwet’s AC1 (95% CI)
Physical incivilities	Property level	Vandalism to private properties	3	2	0.87 (0.75, 0.99)
		Vacant properties	0	0	1.00 (1.00, 1.00)
		Burn out properties	0	1	0.98 (0.93, 1.00)
		Broken windows/doors	0	0	1.00 (1.00, 1.00)
		Abandoned cars	1	1	1.00 (1.00, 1.00)
	Street level	Public area maintenance	24	26	0.93 (0.82, 1.00)
		Stray dogs	2	3	0.88 (0.78, 0.99)
		Derelict land	0	0	1.00 (1.00, 1.00)
		Illegal parking	0	1	0.98 (0.94, 1.00)
		Dog litter in the street	0	0	1.00 (1.00, 1.00)
		Littered street	13	17	0.27 (0.00, 0.58)
		Vandalism to public property	1	1	0.96 (0.89, 1.00)
		Poor path condition	33	30	0.43 (0.16, 0.71)
Territorial functioning	Property level	Low external beautification	5	11	0.42 (0.20, 0.65)
		Low garden maintenance	4	8	0.64 (0.44, 0.84)
		Low property maintenance	11	4	0.39 (0.15, 0.63)
	Street level	No neighbourhood watch signs	33	38	0.83 (0.68, 0.98)
Defensible space	Property level	Low defensible space	4	4	0.80 (0.60, 0.99)
	Street level	Public parking (on street or public court)	5	5	0.66 (0.48, 0.84)
		Intense dense properties	9	9	1.00 (1.00, 1.00)
Natural environment	Property level	No trees in front gardens	6	5	0.91 (0.80, 1.00)
	Street level	Front outlook: green	22	23	0.96 (0.88, 1.00)
		Front outlook: commercial	14	13	0.94 (0.86, 1.00)
		Front outlook: industrial	4	4	1.00 (1.00, 1.00)
		No trees in public space	27	22	0.82 (0.68, 0.96)
		No planted vegetation	22	24	0.83 (0.67, 0.99)
		No green space	21	22	0.96 (0.88, 1.00)
		No recreational space	44	42	0.95 (0.88, 1.00)

Benchmark: Almost perfect (>0.8); Substantial (0.6 ~ 0.8); fair/slight (<0.6).

#### Almost perfect agreement

Twenty-two items were found to have high inter-method reliability and their Gwet’s AC1 coefficient was over 0.8. Six items had 100% agreement. Most of the items regarding defensible space and the natural environment were highly consistent. Rare physical incivilities were also positively associated.

#### Substantial agreement

Two items (garden maintenance and parking arrangement) had an agreement in the substantial range (Gwet’s AC1 coefficient 0.6-0.8).

#### Fair/slight agreement

Fair/slight agreement was found in four items from physical incivilities and territorial functioning. Two were from physical incivilities (littered level of the street and pavement condition) and two from territorial functioning (external beautification and property maintenance).

### The variations between urban and rural areas

A wider range of total scores was found in urban (5.0 ~ 36.0) than rural areas (4.0 ~ 21.5). The postcodes in urban areas were more likely to have a higher proportion of physical incivilities, worse territorial functioning, lower defensible space and a poorer natural environment than rural areas. The analysis of total and domain scores by urban and rural areas is presented in Table 4. In general, the mean differences of the total and domain score were similar and the limits of agreement in rural areas slightly narrower than in urban areas. The magnitude of variation was larger in urban areas (28%) compared to rural areas (16%). Physical incivilities in urban areas had especially broad limits of agreement, ranging approximately from -7 to 7 as well as the highest magnitude of variation (70%).

**Table 4:** **The mean difference and limits of agreement in urban and rural areas**

		Urban			Rural	
Domain	Mean difference (95% CI)	Limits of agreement	Magnitude of variation	Mean difference (95% CI)	Limits of agreement	Magnitude of variation
Total score	0.53 (-1.50, 2.54)	-8.10, 9.15	27%	-0.74 (-1.82, 0.34)	-5.73, 4.25	16%
Physical incivilities	0.10 (-1.54, 1.74)	-6.89, 7.09	70%	-0.52 (-1.45, 0.41)	-4.82, 3.78	43%
Territorial functioning	-0.18 (-0.85, 0.50)	-3.05, 2.70	29%	-0.26 (-0.90, 0.38)	-3.21, 2.69	30%
Defensible space	0.25 (-0.05, 0.55)	-1.03, 1.53	21%	0.13 (-0.07, 0.33)	-0.79, 1.05	15%
Natural environment	0.35 (-0.18, 0.88)	-1.92, 2.62	18%	-0.09 (-0.31, 0.14)	-1.12, 0.94	8%

## Discussion

### Main findings

To our knowledge, this is the first study which has explored the reliability of assessing environmental features associated with mental health and quality of life by visual image audits including urban and rural areas in the UK. Visual image audits using Google Street View were generally consistent with physical audits when considering the magnitude of variation (22%). Little variation was found between the two methods in the domain scores of territorial functioning, defensible space and natural environment. A high magnitude of variation (57%) was found relate to measures of physical incivilities, with broad limits of agreement contributing substantially to the variation in total REAT scores between methods. Nevertheless, most of the items in the REAT showed substantial agreement (Gwet’s AC1 > 0.6) between the two methods and only a few items in the domains of physical incivilities and territorial functioning showed low consistency (Gwet’s AC1 < 0.6). Compared to rural areas, urban areas had wider limits of agreement and a higher magnitude of variation (28%), indicating a lower consistency between the two methods in urban environments.

Compared to other tools designed to assess built environment for physical activity, the REAT particularly focuses on some detailed features in the micro-scale environment. Instead of measuring macro-scale urban design factors, such as street connectivity and land use [15, 18], the REAT items target the physical features related to perceptions of disorder, lack of control and aesthetics, which are important to mental health and quality of life. Although lower agreement in some detailed features, such as litter and street conditions, was found in this study, our findings suggest that visual image assessment could be a useful alternative to physical audits to assess the quality of living environments for mental health and well-being, particularly in large populations where on-foot audits may be hard to conduct. The variation in total score between the physical and visual image audits was generally less than 7, which is not substantial when the range is considered (the magnitude of variation = 22%). However, larger disagreement was found for physical incivilities with a score difference up to 6 in most postcodes (57%). The findings of this study suggest that visual image audits show acceptable reliability in most REAT items and total score but within this individual items and domain scores should be treated with caution. Conducting the REAT assessment through visual image audits can provide a method to detect meaningful differences across areas.

### Limitations

The assessment tool in this study was designed to examine some specific characteristics of built environment in the UK postcodes and measure property and street level features related to mental health and well-being. Since the characteristics of community environments vary considerably in different countries and areas, the validity and reliability of assessment tools and visual image audits may be culture and geography specific. Due to various natural, social and cultural backgrounds, the built environments may differ across areas. Such variation might affect the validity of measurement tools and the ability of visual image audits for environmental assessment in other countries and should be explored further.

This study only included postcodes from one geographical area of England, considered to be a relatively wealthy area in the UK, and less likely to contain poor living environments with high level of disorder. However, there are pockets of deprivation within this area, and the majority of the urban population in England live in cities and towns which are similar in physical characteristics to Cambridge city [19]. Hence, although this study did not include the postcodes in large urban conurbations, the areas surveyed for this research were those typically experienced by more than 60% of the English population [29]. Inter-rater reliability of visual image audits was not addressed in this study as all the assessments were conducted by one assessor. However, since the REAT has been shown to have good reliability in physical audits, it is expected that assessors who are familiar with visual streetscape images could follow the REAT manual and repeat the results of visual image audits appropriately [22].

Although a range of living settings were analysed, the property level characteristics of flats was particularly difficult to assess since the individual properties cannot be separately investigated for either method. A specific measurement method or different scoring system would need to be developed to evaluate the property level conditions for flats in which a substantial proportion of urban populations live. Property level assessment might also be substantially obstructed due to tall walls and trees in both physical and visual image audits.

Some limitations of visual image audits were found in this validation study of the REAT. To conduct property level assessment, it is necessary to identify the houses in the postcode of interest. In some areas, there were no clear house numbers or the numbers were too ambiguous to confirm from the images. This problem can be partially rectified using other information from Royal Mail and property websites to identify approximate areas of postcodes. The measures of physical incivilities and territorial functioning were particularly different between the two methods, and it may be that the resolution of Google Street View images is not sufficient to detect some detailed features, such as property and garden maintenance. The images near the ground are more likely to be distorted because of the higher position of the camera. This might result in lower inter-method reliability for particular items such as pavement conditions and litter on the street.

### Urban and rural areas: changes in community environments

More variation between the two methods was found in urban areas, especially in the domain of physical incivilities. This might be attributed to changes in community environments. Since most of the images of Google were constructed in 2008, the actual interval between physical and visual assessments is nearly four years. Changes in the built environment, especially in urban areas, might lead to greater disparity between what is and what once was. High mobility of residents in urban areas and different occupants of houses may cause variation in findings for the measure of vandalism to private properties could for example be explained caused by such changes. It is noteworthy that graffiti that was found in Cambridge city on the images of 2008 had been removed by the time of the physical audits in 2012. Compared to urban areas, residents in rural areas are expected to be more stable with fewer non-residents passing through [30]. Most of postcodes in rural areas had fewer features of disorder and more consistent results between the two audits.

Some items, such as the level of litter on the street and parking conditions, may vary across the day especially in urban areas. Several images in the Google database were taken in the early morning before refuse collections and the streets were particularly dirty and littered. Similarly, car parking patterns can be different between working and non-working hours.

### Time and potential costs in physical and visual image audits

The findings did not show a considerable difference in terms of the time required for data collection between the two methods but visual image audits are perhaps the most amenable to being speeded up when assessors become more familiar with the process. Based on the cost required for data collection, visual assessment is considered as a less expensive method than physical audits. The travel costs in physical audits can be substantially influenced by study settings and local resources. Mobility in rural areas, where the coverage of public transport is lower, was a major issue in this UK-based study. Although using private vehicles was convenient to finish the assessments in rural areas, it can substantially increase the costs of research. Unpredictable weather and safety issues might also delay the progress of data collection. On the other hand, computer-based data collection in visual image audits requires no resources other than a computer and internet access. Assessors can conduct the assessment in a relatively comfortable and safe environment with more flexible working hours.

### Future research directions

The REAT provides a set of indices to objectively assess the built environment in communities and help identify any the true effect of place on mental health. The results of this study indicate that Google Street View is a feasible tool to assess environmental features related to mental health with fair reliability. Nevertheless, low agreement in some detailed features of disorders (litter, graffiti and abandoned houses) and the conditions of pavements or streets was found in this study. These features are especially difficult to be examined by visual streetscape images. In rural areas, some items, such as graffiti, littered streets and illegal parking, were less likely to be found and might not be so relevant for assessments in rural settings. Future research can build on these results to adjust existing instruments for visual image audits or develop specific tools for remote assessment in different areas.

Conducting the REAT through streetscape images substantially sidesteps the disadvantages in traditional physical audits and provides the opportunity to investigate the influence of specific environmental features on mental health in large populations. Since the reliability of physical and visual image audits has been tested and verified in this study, the method of visual assessment could be applied to other settings to measure environmental features for study their associate with mental health and quality of life.

## Conclusion

In this paper, we present a novel measurement method using lower resource intensity than physically visiting localities. The findings suggest that Google Street View has acceptable reliability to assess environmental features related to mental health when compared to the REAT assessment tool and a score difference up to 22% for the total score. Although wider limits of agreement and larger variation in domain scores need to be treated with caution, visual image audits have the opportunity to overcome some of the disadvantages in traditional physical audits and provide insights into the potential influences of the built environment on mental health in large populations where on-foot audits may be impractical.

## Abbreviations

REAT: Residential environmental assessment tool.
